# Supplementary material for: TAZ Controls Helicobacter pylori-Induced Epithelial–Mesenchymal Transition and Cancer Stem Cell-Like Invasive and Tumorigenic Properties
Source: Cells. 2020 Jun 13;9(6):1462. doi: 10.3390/cells9061462 (PMC7348942; doi:10.3390/cells9061462)
Supplement: Supplementary file 1 [file cells-09-01462-s001.pdf]

cells MDPI

Supplementary Materials: Supplementary Figure S1 and Supplementary Figure S2 and their corresponding figure legends.

Supplementary Figure S1:

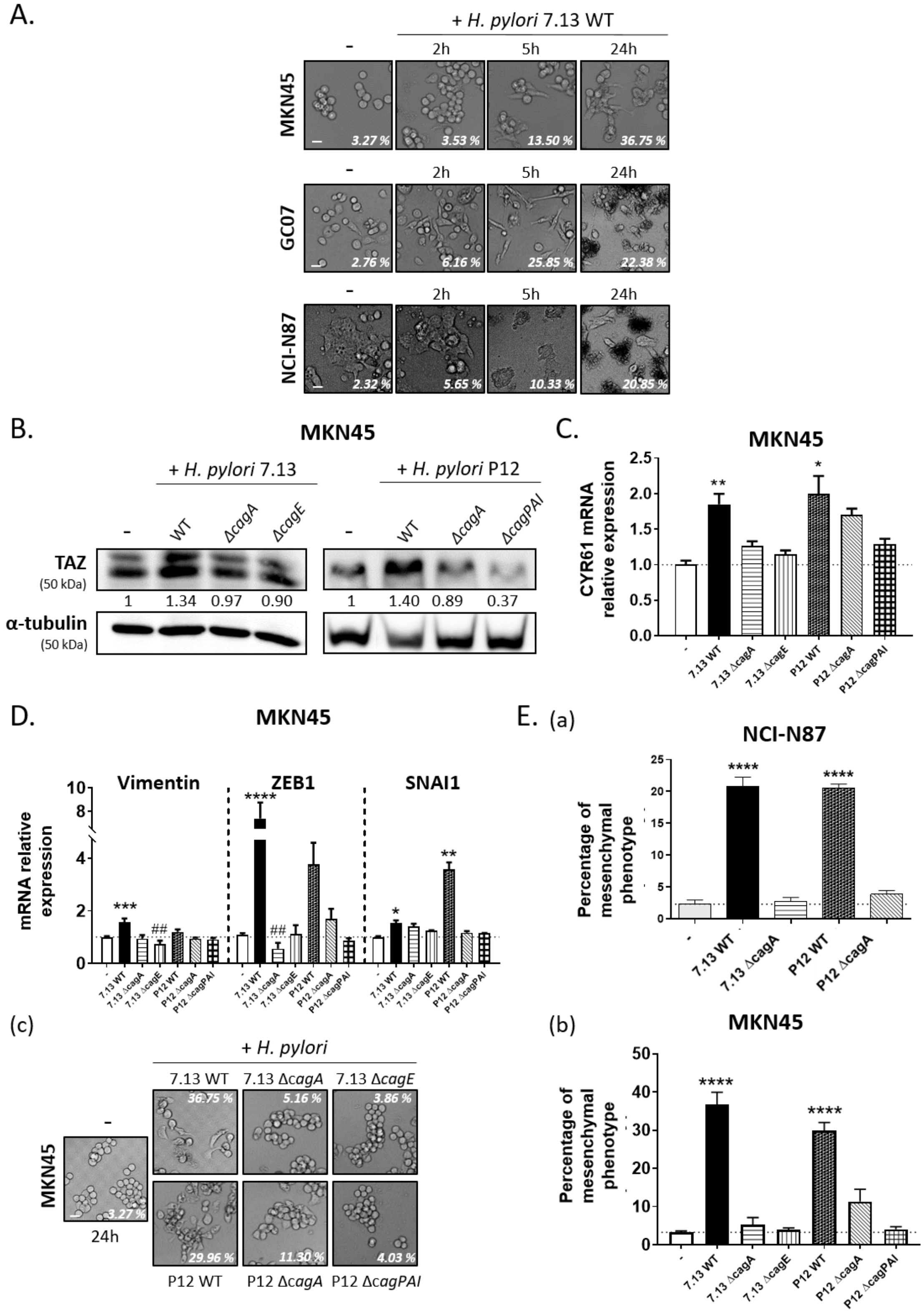

Figure S1. ***H. pylori*** **virulence factor CagA is implicated in TAZ overexpression and EMT. (A)** Phase-contrast images of gastric epithelial cells during a time-course experiment of 2, 5 and 24 h after *H. pylori* 7.13 WT strain infection. Percentages indicated at the bottom right in each image correspond to the number of cells exhibiting a mesenchymal phenotype. **(B-E)** Investigation of the role of CagA and the T4SS in *H. pylori*-induced TAZ overexpression. Co-culture of MKN45 cells, 7.13 and P12 *H. pylori* strains either WT or invalidated for CagA (Δ*cag*A) or the T4SS (Δ*cag*E and Δ*cag*PAI) after 24 h of infection. **(B)** Expression of TAZ evaluated by Western blot. **(C)** CYR61 relative mRNA expression assessed by RTqPCR. **(D)** Relative mRNA expression of Vimentin, ZEB1 and SNAI1 assessed by RTqPCR. **(E)** Percentage of cells with the mesenchymal "hummingbird" phenotype in NCI-N87 cells **(a)** and in MKN45 cells **(b)**. **(c)** Phase-contrast images of MKN45 infected with the different *H. pylori* strains. Percentages at the bottom right of each image correspond to the number of cells with a mesenchymal phenotype. **(A, E)** Scale bars, 10 µm. **(B-E)** Bars represent means ± SEM, n=3, *$p<0.05$ *vs* uninfected control "-". # *vs* respective WT *H. pylori* strain. **(C-D)** Kruskal-Wallis with Dunn's post-test. **(E)** ANOVA test.

Supplementary Figure S2:

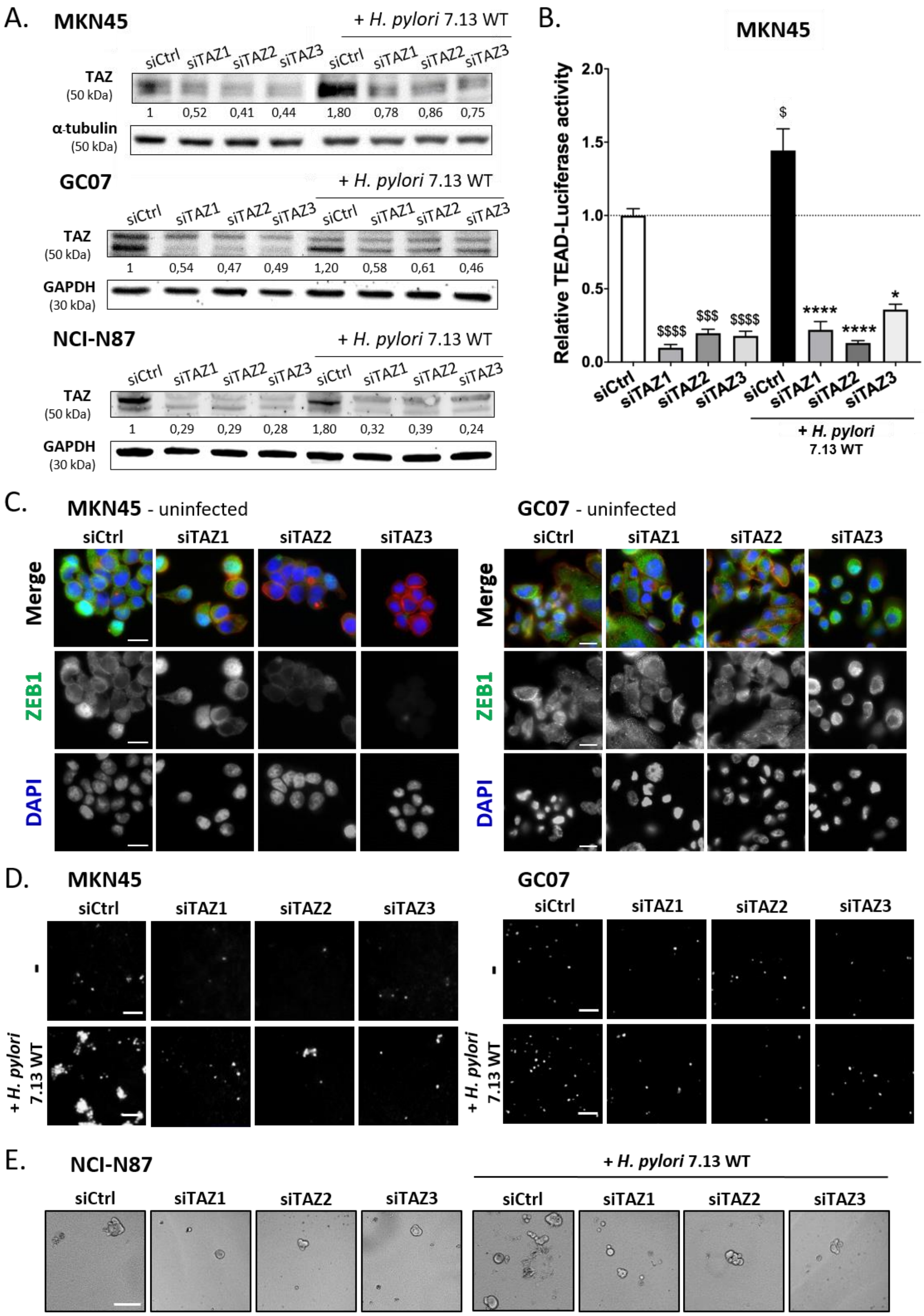

Figure S2. Effect of TAZ silencing by siRNA on TAZ expression, TEAD-luciferase activity, ZEB1 expression, invasive and tumourigenic cells properties. (A) Validation of TAZ knock-down after siRNA silencing and *H. pylori* infection or not in MKN45, GC07 and NCI-N87 cells assessed by Western blot. Number represents the quantification of the relative TAZ protein expression compared to uninfected siCtrl cells and normalised to GAPDH or α-tubulin protein expression. **(B)** Validation

of siTAZ silencing efficiency assessed by relative TEAD-luciferase reporter activity performed in MKN45 cells. Bars represent means ± SEM of fold changes relative to uninfected cells. n=4, $ *vs* uninfected siCtrl and * *vs H. pylori*-infected siCtrl. Kruskal-Wallis test with Dunn's post-test. **(C)** Immunofluorescence of ZEB1 (in green), actin cytoskeleton stained by phalloidin (in red) and nuclei stained with DAPI (in blue) performed in MKN45 and GC07 siTAZ-transfected cells. Scale bars, 10 μm. (D) Representative images illustrating invading cells of Figure 4D. MKN45 and GC07 cells were transfected with siCtrl or 3 different siTAZ and infected with *H. pylori* for 24 h. DAPI positive staining of invasive cells was counted 18 h after seeding on type I collagen coated inserts. Scale bars, 100 μm. (E) Representative images illustrating NCI-N87 tumorspheres of Figure 4F. NCI-N87 cells were seeded in non-adherent conditions during 7 days after being transfected with siCtrl or 3 different siTAZ and infected with *H. pylori* during 24 h. Scale bars, 100 μm.

© 2020 by the authors. Licensee MDPI, Basel, Switzerland. This article is an open access article distributed under the terms and conditions of the Creative Commons Attribution (CC BY) license (http://creativecommons.org/licenses/by/4.0/).
